# Quantitative Determination of Minerals and Toxic Elements Content in Tropical and Subtropical Fruits by Microwave-Assisted Digestion and ICP-OES

**DOI:** 10.1007/s12011-024-04265-7

**Published:** 2024-06-14

**Authors:** Fahad AlJuhaimi, Duygu Akçay Kulluk, Fatma Gökmen Yılmaz, Isam Ali Mohamed Ahmed, Mehmet Musa Özcan, Zainab Albakry

**Affiliations:** 1 Department of Food Science & Nutrition, College of Food and Agricultural Sciences, King Saud University, Riyadh, Saudi Arabia; 2 Department of Soil Science and Plant Nutrition, Faculty of Agriculture, Selcuk University, 42031 Konya, Turkey; 3 Department of Food Engineering, Faculty of Agriculture, Selcuk University, 42031 Konya, Turkey; 4 College of Ocean Food and Biological Engineering, Jimei University, Xiamen, 361021 China

**Keywords:** Tropic and subtropical fruits, macro and micro elements, toxic elements, Pearson analysis analysis, ICP-OES

## Abstract

In the presented study, 15 tropical and subtropical fruits were studied for their mineral composition ranging from trace to major elements by ICP-OES after microwave digestion. The moisture amounts were assigned to be between 21.90 (tamarind) and 95.66% (pepino). The differences between the macroelement quantities of the fruits were established to be statistically significant (p<0.01). P and K quantities of fruits were displayed to be between 53.40 (pepino) and 927.74 mg/kg (tamarind) to 720.27 (pepino) and 13441.12 mg/kg (tamarind), respectively. While Ca quantities of fruits vary between 123.71 (pineapple) and 1519.76 mg/kg (blood orange), Mg quantities of fruits were established to be between 78.66 (pepino) and 875.02 mg/kg (tamarind). In general, the lowest macroelement quantities were determined in pepino fruit, but the highest P and K contents were determined in Gooseberry and Tamarind fruits, respectively. The microelement amounts of the fruits were established to be at very low levels compared to the macroelement contents. In general, the most abundant element in fruits was Fe, followed by Zn, Cu, Mn and B in decreasing order. In general, heavy metal quantities of fruits were detected at very low levels (except As and Ba). As and Ba quantities of fruits were assigned to be between 0.972 μg/g (mandarin) and 5.86 (kiwi) to 0.103 (pineapple) and 4.08 (avocado), respectively. As with macro and microelements, results regarding heavy metal concentrations varied depending on fruit types.

**Graphical Abstract:**

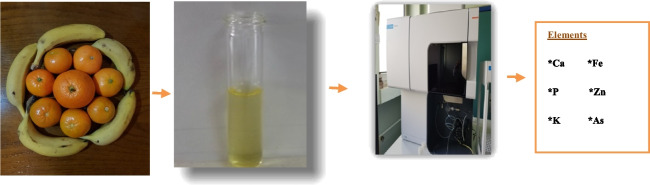

## Introduction

Tropical and subtropical fruits, which contain nutrients, minerals, trace elements, vitamins, antioxidant compounds and phytochemicals that are important for human health, are preferred as medicinal foods not only due to their special taste and organoleptic properties such as mouthfeel, but also especially due to their high nutritional values. and this makes them recommended for treatment purposes [1–4]. Calcium, potassium, iron, phosphorus, magnesium, zinc and some trace elements, which constitute the most important minerals of fruits, play a vital role in metabolic pathways in humans, and fruits are a good source of basic mineral elements required for many physiological and metabolic reactions involved in maintaining health [5–8]. Although the trace mineral density of fruits varies depending on genetics, weather, soil and harvest maturity stage, climatic conditions caused by rain, fog, light and temperature reduce the mineral content in plants and then further losses occur due to inappropriate storage conditions. Also, trace elements in the soil and their deficiency will negatively affect the composition of the crop. The elements play an important role in biological systems, they are necessary for maintaining of life, normal physiological functioning (e.g. body pH and osmotic balance), growth, development and function of human cells as well trace elements also serve as coenzymes that regulate metabolic reactions [7]. Minerals, which are basic regulators of health protection and various physiological and metabolic reactions, help approximately one-third of all human proteins to function properly [9]. Since the human body cannot synthesize mineral nutrients, fruits and vegetables are needed to provide regular amounts of the diet. Fruits such as banana, kiwi, mango and pineapple, which contribute to the mineral nutrients necessary to maintain health, should be included in the daily diet [10, 11]. Changes in maturity, soil structure and agricultural applications can affect mineral concentrations in plants, causing different mineral nutrients to appear in plants [9]. In addition to determining the concentrations in fruits and vegetables to ensure that the mineral levels in the diet are at the desired level, it is important for human health, the toxicity of elements such as Al, As, Cd, and Pb [12]. Consumers' awareness of balanced nutrition makes it necessary to determine the nutritional composition (vitamins and minerals) of normally consumed fruits [13]. Fruits are effective in the metabolism of many functions in the human organism because they contain vitamins, dietary fibers and essential mineral substances in various amounts and quality, and studies on the mineral composition of tropical fruits need to be intensified because they affect the functional performance of metabolism ([13]; Anna [14]). Considering that people are increasingly aware of the need for a balanced diet, the aim of this study was to determine the mineral content of 15 different fruits grown in tropical and subtropical climate conditions using acidic microwave-assisted digestion followed by ICP-OES. Comparing metal contents in fruits leads to evaluation of the elemental differences between fruits and useful information for nutritional science. The purpose of the paper was to investigate macro-elements (P, K, Ca, Mg, S), trace elements (Fe, Zn, Cu, Mn, B) and non-essential and toxic element contents (As, Ba, Cd, Co, Cr,Mo, Ni, Se and Pb) of some tropic and subtropic fruits.

## Materials and methods

### Materials and sample preparation

Tropical and subtropical fruits used in this study were purchased from local markets in Konya Turkey (2023). Each fruit was individually chopped into thin slices (about 2-3 mm) with a stainless steel knife. These samples were dried at 70 ^o^C for 24 hours. The dried samples were ground in a laboratory mill and homogenized in a metal free mortar and stored in a dry and dark place at ambient temperature prior to digestion. HNO_3_ and H_2_O_2_ were analytical grade and purchased from Merck (Munich, Germany) Table 1.

**Table 1:** Tropical and subtropcal fruits used in this study

**Fruits**	**Scientifc names for fruits**
Pineapple	*Ananas comosus*
Cape Gooseberry	*Peruvian groundcherry*
Avocado	*Persea americana*
Tamarindus İndica	*Tamarindus indica*
Dragon Fruit	*Hylocereus spp.*
Grapefruit	*Citrus paradisi*
Blood Orange	*Citrus sinensis*
Kiwi	*Actinidia deliciosa*
Kumquat	*Citrus fortunella*
Mandarin	*Citrus reticulata*
Mango	*Mangifera indica*
Banana	*Musa cavendishii*
Pepino	*Solanum muricatum*
Orange	*Citrus aurantium*
Bilberry	*Vaccinium myrtillus*

The places where the fruit samples were purchased from local markets in Konya (local markets in Konya (37°52′N 32°29′E)

## Method

### Determination of moisture

The moisture results of the fruits were detected at 70 °C/48 hours using an oven till a constant weight [15].

### Macro, micro and toxic element contents of tropical and subtropical fruits

#### Digestion

After 0.2 g fruit samples were inginerated in a microwave device (CEM mars Xpress 6 One Touch Model, USA) at 210 °C and 200 PSI pressure in 5 ml of concentrated HNO_3_ and 2 ml of H_2_O_2_ (30% *w/v*), the volumes of the dissolved samples were completed to 20 ml with deionized water.

#### Analysis

Macro- and microelement content was determined using a inductively coupled plasma optical emission spectroscopy (ICP- OES (Agilent-5110)) operated in simultaneous mode according to Tošic et al. [16]. The standard solutions were prepared by dilution of a multi-element standard stock solution (1000 mg/L) with 1% *w/w* HNO_3_. Blank solutions were prepared in the same medium.

- ***Working conditions of ICP-OES:***
- Instrument :ICP-OES
- RF Power : 0.7-1.5 kw (1.2-1.3 kw for Axial)
- Plasma gas flow rate (Ar) : 10.5-15 L/min. (radial) 15 “ (Axial)
- Auxilary gas flow rate (Ar) :1.5 “
- Viewing height : 5-12 mm
- Copy and reading time :1-5 s (max.60 s)
- Copy time : 3 s (max. 100 s)
- Wavelengths, detection LOD (mg/kg), Limit of Quantification LOQ (mg/kg), Coefficient of determination (R^2^) of elements analyzed with ICP-OES:

**Table:** 

Elements	Wavelengths (nm)	Detection LOD (mg/kg)	Limit of Quantification LOQ (mg/kg),	Coefficient of determination (R^2^)
P	213.618	4.346591	14.48864	0.999
K	766.491	131.6843	438.9477	0.999
Ca	317.933	259.2863	864.2877	0.999
Mg	279.553	58.98845	196.6282	0.999
S	181.972	0.013909	0.046364	0.999
Fe	238.204	1.411773	4.705909	0.999
Zn	213.857	0.208636	0.695455	0.999
Cu	327.395	0.027818	0.092727	0.999
Mn	257.610	0.424227	1.414091	0.999
B	249.772	0.118227	0.394091	0.999
As	175.800	0.729991	2.433303	0.999
Ba	614.171	43.81364	146.0455	0.999
Cd	214.439	0.006955	0.023182	0.999
Co	238.892	0.006955	0.023182	0.999
Cr	267.716	0.000695	0.002318	0.999
Ni	231.604	1.578189	5.260631	0.999
Pb	220.353	5.276961	17.58987	0.999
Se	203.985	0.037555	0.125182	0.999
Mo	202.032	0.041727	0.139091	0.999

### Statistical analysis

The JMP statistical program was used for the statistical analysis of findings obtained. Statistically differences were established by the analysis of variance (ANOVA) procedure in all data (p<0.01. All measurements were carried out in triplicate, and presented as mean±standard deviation (SD). [17]. In order to examine the correlation between nutrient element contents of tropical and subtropical fruits, Principal Component Analysis (PCA) was applied [18].

## Results and Discussion

### Macroelement contents of some topical and subtropical fruits

Moisture and macroelement quantities of some tropical and subtropical fruits are assigned in Table 2. Macroelement and moisture quantities varied depending on fruit types. The moisture amount of the fruits were depicted to be between 21.90 (tamarind) and 95.66% (pepino). The differences between the macroelement quantities of the fruits were established to be statistically significant changes (p<0.01). P and K quantities of fruits were evaluated to be between 53.40 (pepino) and 927.74 mg/kg (tamarind) to 720.27 (pepino) and 13441.12 mg/kg (tamarind), respectively. While Ca quantities of fruits vary between 123.71 (pineapple) and 1519.76 mg/kg (blood orange), Mg quantities of fruits were established to be between 78.66 (pepino) and 875.02 mg/kg (tamarind). The lowest Ca content among fruits was detected in Pineapple and Pepino fruits. In general, the lowest macro element contents were determined in Pepino fruit, but the highest P and K contents were determined in gooseberry and Tamarind fruits, respectively. It was determined that the fruits used in this study contained the highest amount of K, followed by P, Ca and Mg in decreasing order. The P quantities of Gooseberry and Tamarind fruits were established to be higher than those of other fruits. In addition, the highest P, K and Mg were detected in Tamarind fruit. Calcium-rich fruits are gooseberry, avocado, tamarind and blood orange. In addition, it is understood that tamarind fruit is rich in element K (13441.12 mg/kg). It is thought that the structure and nutrient element content of the soil where the fruits grow, the root structure, the transport of elements from the soil to the plant organs and the conditions will be among the factors affecting the element concentrations of the fruits ([9]; Habte et al. [19–21]. It was observed that the macroelement contents of the fruits differed from the literature data [20, 22, 23]. It is thought that factors such as harvest time, growing conditions, fruit type and genetic structure may be effective in these differences.

**Table 2:** Moisture (%) and macro element contents of tropical and subtropical fruit varieties (mg/kg)

**Samples**	**Moisture**	**P**	**K**	**Ca**	**Mg**
**Pineapple**	90.07±0.425 b*	55.07±5.92 **ıj**	1080.97±116.24 **h**	123.71±11.72 **ı**	153.68±19.80 **e-ı**
**Cape Gooseberry**	80.55±1.21 **ı**	792.94±192.23 **b**	4848.24±173.84 **b**	1072.84±15.80 **cd**	322.64±78.02 **c**
**Avocado**	85.78±0.050 **d-g**	373.32±3.61 **cd**	2887.32±37.10 **cd**	1323.72±221.82 **ab**	272.65±43.25 **cd**
**Tamarindus İndica**	21.90±3.40 **l**	927.74±80.69 **b**	13441.12±104.62 **a**	1283.56±265.77 **bc**	875.02±90.01 **a**
**Dragon Fruit**	86.17±0.315 **cde**	327.36±3.17 **de**	2640.03±328.43 **de**	608.38±71.80 **fg**	334.02±54.98 **bc**
**Grapefruit**	87.62±0.100 **c**	346.81±43.56 **cde**	1419.96±151.25 **g**	470.88±50.14 **gh**	109.20±15.87 **g-j**
**Blood Orange**	82.97±0.745 **h**	286.33±10.09 **d-g**	2001.79±72.57 **f**	1519.76±307.45 **a**	131.59±1.99 **f-j**
**Kiwi**	84.99±0.510 **efg**	443.14±11.32 **c**	2524.99±27.32 **e**	652.20±107.81 **efg**	171.82±35.88 **efg**
**Marumi**	70.94±0.670 **k**	293.71±24.59 **d-g**	2061.37±428.82 **f**	979.22±36.75 **d**	211.61±22.75 **de**
**Mandarin**	87.0±0.275 **cd**	253.94±16.11 **e-h**	1577.19±190.73 **g**	527.78±109.05 **fgh**	164.04±18.93 **e-h**
**Mango**	84.14±0.060 **gh**	215.46±82.02 **gh**	2097.84±392.07 **f**	734.17±153.86 **ef**	182.96±16.65 **ef**
**Banana**	78.66±0.285 **j**	319.04±46.70 **def**	3187.85±54.41 **c**	505.44±8.93 **fgh**	393.72±33.90 **b**
**Pepino**	95.66±0.290 **a**	53.47±7.56 **j**	720.27±44.80 **ı**	356.99±34.30 **hı**	78.66±5.31 **j**
**Orange**	86.15±0.050 **c-f**	220.25±16.11 **fgh**	1657.08±156.25 **g**	878.87±109.97 **de**	94.63±1.99 **ıj**
**Bilberry**	84.48±0.610 **fgh**	155.33±12.87 **hı**	1078.61±18.65 **h**	528.66±117.58 **fgh**	104.52±12.84 **hıj**

*p<0.01

### Microelement quantities of some topical and subtropical fruits

Microelement distributions in fruits are shown in Table 3. As with macroelement contents, microelement contents of fruits varied depending on fruit types. The changes between the microelement quantities of the fruits were evaluated to be statistically significant. The microelement contents of the fruits were found to be at very low levels compared to the macroelement contents. In general, the most abundant element in fruits was Fe, followed by Zn, Cu, Mn and B in decreasing order. Fe and Zn quantities of fruits were detected to be between 3.90 (mandarin) and 22.71 mg/kg (gooseberry) to 0.721 (pepino) and 8.67 mg/kg (pineapple), respectively. The highest levels of Cu (9.07), Mn (9.31) and B (14.42 mg/kg) were detected in Tamarind, avocado and Tamarind fruits, respectively. In addition, the lowest Mn (0.480) and B (0.249 mg/kg) were determined in pepino fruit. In general, the mineral quantities of Pepino fruit were found to be lower levels than those of other fruits. Results showed some changes compared with literature values. Differences in the distribution of microelements in fruits may be caused by rainfall, fertilization types and forms, genetic structure, climatic and agronomic activities and some analytical conditions.

**Table 3:** Micro element contents of some tropical and subtropical fruit varieties (mg/kg)

**Samples**	**Fe**	**Zn**	**Cu**	**Mn**	**B**
**Pineapple**	5.31±0.661 fg*	8.67±0.194 **a**	0.451±0.066 **g**	2.84±0.250 **bc**	0.348±0.038 **ı**
**Cape Gooseberry**	22.71±1.01 **a**	3.95±0.206 **b**	5.87±1.38 **c**	2.07±0.570 **d**	1.57±0.070 **efg**
**Avocado**	22.62±2.57 **a**	3.61±0.268 **b**	7.22±0.535 **b**	9.31±0.391 **a**	2.08±0.145 **cde**
**Tamarindus İndica**	14.64±2.53 **bc**	4.03±0.281 **b**	9.07±1.27 **a**	2.38±0.200 **cd**	14.42±1.50 **a**
**Dragon Fruit**	12.66±1.23 **c**	4.06±0.571 **b**	2.42±1.06 **ef**	2.03±0.286 **d**	2.32±0.005 **bcd**
**Grapefruit**	6.45±0.392 **fg**	1.17±0.230 **ef**	1.61±0.486 **ef**	0.374±0.092 **gh**	0.934±0.003 **ghı**
**Blood Orange**	7.93±1.49 **def**	1.13±0.213 **ef**	1.98±0.373 **ef**	0.681±0.008 **fgh**	2.91±0.546 **b**
**Kiwi**	9.09±1.74 **de**	1.52±0.411 **de**	2.55±0.546 **e**	2.50±0.175 **bcd**	1.33±0.217 **fg**
**Marumi**	6.93±0.649 **efg**	1.17±0.053 **ef**	1.96±0.044 **ef**	0.969±0.078 **ef**	2.71±0.257 **bc**
**Mandarin**	3.90±0.844 **h**	0.731±0.082 **f**	1.42±0.211 **fg**	0.274±0.003 **h**	1.74±0.161 **def**
**Mango**	15.28±0.346 **b**	1.76±0.455 **cd**	3.95±1.02 **d**	0.878±0.227 **efg**	0.504±0.049 **hı**
**Banana**	9.00±0.116 **de**	2.13±0.080 **c**	2.30±0.091 **ef**	1.38±0.008 **e**	1.10±0.006 **fgh**
**Pepino**	5.04±0.498 **gh**	0.721±0.071 **f**	1.44±0.142 **efg**	0.480±0.047 **fgh**	0.240±0.024 **ı**
**Orange**	6.10±1.05 **fgh**	1.35±0.219 **de**	1.39±0.010 **fg**	0.491±0.032 **fgh**	1.31±0.078 **fg**
**Bilberry**	9.94±1.87 **d**	1.55±0.266 **de**	2.26±0.240 **ef**	2.95±0.904 **b**	1.08±0.054 **fgh**
**Samples**	**Mo**	**Ni**		**Pb**	**Se**
**Pineapple**	0.074±0.0032 gh*	0.075±0.0013 **e**		0.172±0.0077 **def**	0.371±0.012 **e**
**Cape Gooseberry**	0.357±0.042 **a**	0.038±0.002 **gh**		0.399±0.006 **bc**	0.156±0.0041 **g**
**Avocado**	0.297±0.0059 **b**	0.177±0.026 **c**		0.724±0.023 **a**	1.19±0.0026 **b**
**Tamarindus İndica**	0.221±0.0052 **c**	0.065±0.0033 **ef**		0.380±0.0073 **c**	2.15±0.103 **a**
**Dragon Fruit**	0.244±0.0018 **c**	0.217±0.0060 **b**		0.073±0.0009 **gh**	0.108±0.0031 **h**
**Grapefruit**	0.060±0.0068 **h**	0.0608±0.011 **ef**		0.108±0.0065 **fgh**	0.424±0.012 **d**
**Blood Orange**	0.057±0.0004 **h**	0.023±0.0007 **hıj**		0.477±0.0026 **b**	0.471±0.016 **cd**
**Kiwi**	0.086±0.0046 **fg**	0.112±0.0023 **d**		0.234±0.0039 **d**	0.223±0.0060 **f**
**Marumi**	0.104±0.012 **ef**	0.451±0.0223 **a**		0.149±0.0011 **efg**	0.028±0.0030 **ı**
**Mandarin**	0.032±0.0059 **ı**	0.064±0.0013 **ef**		0.061±0.0014 **h**	0.083±0.0031 **h**
**Mango**	0.129±0.027 **d**	0.051±0.0014 **fg**		0.366±0.0077 **c**	0.257±0.0187 **f**
**Banana**	0.124±0.0054 **de**	0.033±0.0030 **hı**		0.371±0.193 **c**	0.495±0.0086 **c**
**Pepino**	0.088±0.0029 **fg**	0.026±0.0002 **hıj**		0.231±0.017 **de**	0.071±0.0003 **hı**
**Orange**	0.095±0.0023 **fg**	0.016±0.0011 **j**		0.153±0.0124 **d-g**	0.253±0.0002 **f**
**Bilberry**	0.080±0.0010 **gh**	0.021±0.0022 **ıj**		0.057±0.0006 **h**	0.225±0.0126 **f**

*p<0.01

### Toxic element content of some topical and subtropical fruits

Toxic element contents of some fruits grown in tropical and subtropical climates are depicted in Table 4. As and Ba quantities of fruits were partially higher than other elements. In general, toxic element amounts of fruits were detected at very low levels (except As and Ba). As with macro and microelements, results regarding heavy metal concentrations varied depending on fruit types. As and Ba amounts of fruit samples were assessed to be between 0.972 μg/g (mandarin) and 5.86 (kiwi) to 0.103 (pineapple) and 4.08 (avocado), respectively. As quantities (4.13-4.88 μg/g) of dragon, grapefruit, blood orange and banana fruits were found to be very close to each other. Except for Gooseberry, Tamarind and Bilberry fruits (2.05-2.94 μg/g), the Ba quantities of other fruits were determined below 2.05 μg/g. Cd and Co amounts of fruits were assigned to be between 0.0020 (marumi) and 0.032 μg/g (gooseberry) to 0.0004 (marumi) and 0.019 μg/g (Tamarind), respectively. In addition, the lowest and highest amount of Cr (0.047 and 0.319, respectively) were found in Mandarin and Gooseberry fruits. Also, Mo and Ni content of fruit samples were established to be between 0.032 (Mandarin) and 0.356 μg/g (gooseberry) to 0.016 (orange) and 0.451 μg/g (marumi), respectively. Lead (Pb) quantities of fruits were assigned to be between 0.057 (Bilberry) and 0.724 μg/g (Avocado). Se amounts of fruit samples changed between 0.028 (Marumi) and 2.15 μg/g (Tamarind). Another fruit with high Se content (1.19 μg/g) was avocado. Se quantities of other fruits were determined below 1.19 μg/g. Contamination of these toxic elements to fruits may be caused by the soil and irrigation water, as well as the atmosphere of the industrial zone, areas with heavy vehicle traffic, packaging of the fruits, transportation and storage methods, and marketing processes. The concentrations of macro-, micro- and toxic elements in the studied fruits showed significant differences depending on the fruit types. These differences may possibly be due to soil structure, harvest time, climatic conditions, irrigation water’s contamination, industrial area and agricultural activities applied. Narain et al. [23] determined the maximum and minimum average K (95.13–270.4), Ca (10.57–75.29), Zn (0.466–1.611) and Mn (0.035–1.902 μg/g) values in citrus fruits. Our findings were low compared to the Zn, Mn, Ca and K results assessed by Narain et al. [23] in kumquat fruit. The mean values of some essential element from tropical fruits were determined and reported in literature. Ca and Mg amounts of fruits changed between 59.1 (banana) and and 358 mg/kg (kiwi) to 92 (lemon) and 287 mg/kg (banana), respectively [20]. Ca quantities of some tropical fruits were reported to be between 400 and 1.200 mg/100g [22]. Burguera et al. [22] assessed the Mn quantities of eight fruits grown in tropical regions between 0.9 and 2.0 mg 100/g, and they determined the highest Mn value (2.0 mg 100/g) in the "macaúba" fruit. Concentrations of essential elements have been reported to vary significantly among tropical fruits, with Mn (0.027–13.2 μg/g) and Zn (0.514–2.20 μg/g) content [21]. In addition, Co (0.002–0.005 μg/g) contents were reported to be at very low levels [21]. Elemental contents of fruit samples showed partial differences with the results of tropic and semitropic fruits by Narain et al. [23], Habte et al. [21] and Cozma et al. [20]. These differences may probably be due to climatic factors, irrigation and fertilization, and environmental factors. While Fe content in tropical fruits vary between 1.12 (banana) and 3.26 mg/kg (pomelo), Mn amount in tropic fruits were determined to be between 0.27 (orange) and 7.12 mg/kg (Pineapple) [20]. Zn and Cu quantities of tropic fruits were established to be between 0.75 (Mandarin) and 1.53 mg/kg (banana) to 0.45 (grapefruit and 1.21 mg/kg (banana), respectively [20]. It has been reported that Zn (Zn ^2+^) participates in the formation of chlorophyll in plants and activates some enzymes, as well as contributing to the protection of the health of the reproductive and immune systems [20]. The concentrations of microelements in tropical fruits differ between 0.9 to 2.0 mg/100g for Mn, 3.9 to 11.4 mg/100g for Fe, 0.5 to 1.0 mg/100g for Cu, 0.6 to 1.5 mg/100g for, Zn [13]. Some tropic and semitropic fruits studied on elements contained 0.0108-0.0156 mg/kg Co, 0.066-0.119 Fe, 0.0051-0.0117 Mn, 0.002-0.012 Ni and 0.0276-0.0624 Zn [7]. The low levels of toxic element amounts in our findings were similar to the literature values. The amounts of As and Cd elements (μg/g) of some tropical and subtropical fruits vary between 0.001 (kiwi) and 0.003 (mango). In addition, while Hg contents of these tropic fruits change 0.0004 (pineapple) to 0.002 (banana), Pb quantities were established 0.005 (kiwi) to 0.013 (mango) [21]. According to Habte et al. [21], the concentrations of toxic elements (Cd, Co, Cr, Mo, Ni, Pb and Se) were found at very low levels. Our results were higher than those of Habte et al. [21]. When our findings regarding the macro-, micro- and toxic element quantities of tropical and subtropical fruits were compared with results of [22]), Narain et al. [23], Habte et al. [21] and Cozma et al. [20], differences were detected. Literature data show significantly higher values than the obtained results. The difference in results may be due to the difference in the fruits used, harvest time, transfer procedures and applied analytical procedures.

**Table 4:** Toxic element contents of tropical and subtropical fruit varieties (**μg/ g)**

**Samples**	**As**	**Ba**	**Cd**	**Co**	**Cr**
**Pineapple**	2.91±0.642 ef*	0.103±0.00027 **k**	0.0056±0.0004 **g**	0.0050±0.0002 **e**	0.082±0.0005 **f**
**Cape Gooseberry**	3.05±0.507 **e**	2.31±0.070 **c**	0.032±0.0023 **a**	0.0065±0.0001 **d**	0.319±0.006 **a**
**Avocado**	3.22±0.298 **de**	4.08±0.054 **a**	0.014±0.0005 **c**	0.010±0.0002 **c**	0.164±0.010 **c**
**Tamarindus İndica**	2.29±0.584 **f**	2.94±0.250 **b**	0.025±0.0014 **b**	0.019±0.0023 **a**	0.203±0.0051 **b**
**Dragon Fruit**	4.38±0.485 **bc**	1.65±0.137 **e**	0.013±0.0014 **c**	0.0050±0.0003 **e**	0.128±0.0047 **d**
**Grapefruit**	4.52±0.107 **bc**	0.852±0.031 **hıj**	0.0053±0.0008 **g**	0.0023±0.00011 **g**	0.073±0.0034 **fg**
**Blood Orange**	4.13±0.137 **c**	1.10±0.135 **gh**	0.0094±0.0004 **e**	0.0035±0.0002 **f**	0.136±0.019 **d**
**Kiwi**	5.86±0.412 **a**	1.38±0.278 **ef**	0.0094±0.0003 **e**	0.0113±0.0004 **b**	0.065±0.0089 **gh**
**Marumi**	2.30±0.423 **f**	1.28±0.064 **fg**	0.0020±0.0002 **ı**	0.0004±0.0001 **h**	0.107±0.0027 **e**
**Mandarin**	0.972±0.104 **g**	0.644±0.045 **j**	0.0023±0.0004 **ı**	0.0006±0.00005 **h**	0.047±0.0025 **ı**
**Mango**	2.29±0.284 **f**	1.64±0.338 **e**	0.0044±0.0011 **gh**	0.0031±0.0002 **fg**	0.138±0.0019 **d**
**Banana**	4.88±0.517 **b**	0.974±0.050 **hı**	0.0031±0.0003 **hı**	0.0022±0.0002 **g**	0.163±0.0187 **c**
**Pepino**	2.89±0.163 **ef**	0.736±0.073 **ıj**	0.0116±0.0010 **d**	0.0025±0.0001 **fg**	0.057±0.0072 **hı**
**Orange**	1.49±0.410 **g**	0.833±0.035 **ıj**	0.0047±0.0002 **g**	0.0065±0.0004 **d**	0.059±0.0042 **ghı**
**Bilberry**	3.84±0.484 **cd**	2.05±0.243 **d**	0.0078±0.0001 **f**	0.0103±0.0011 **bc**	0.051±0.0049 **hı**

*p<0.01

### The Pearson correlation analysis

**Fig. 1:**
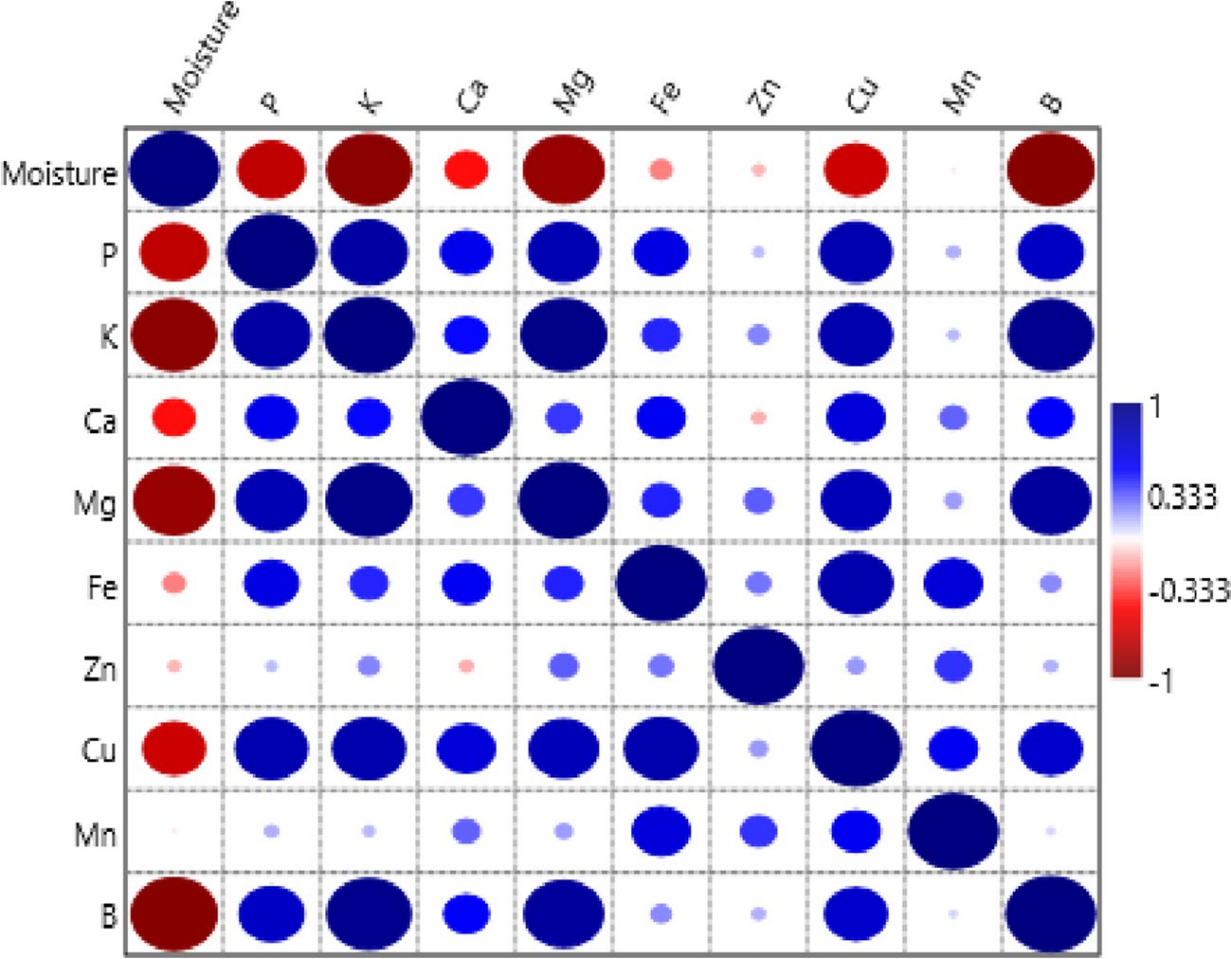
Pearson correlation (r) between macro (P, K, Ca and Mg) and micro (Fe, Zn, Cu, Mn and B) nutrient contents and moisture contents of tropical fruit varieties

**Fig. 2:**
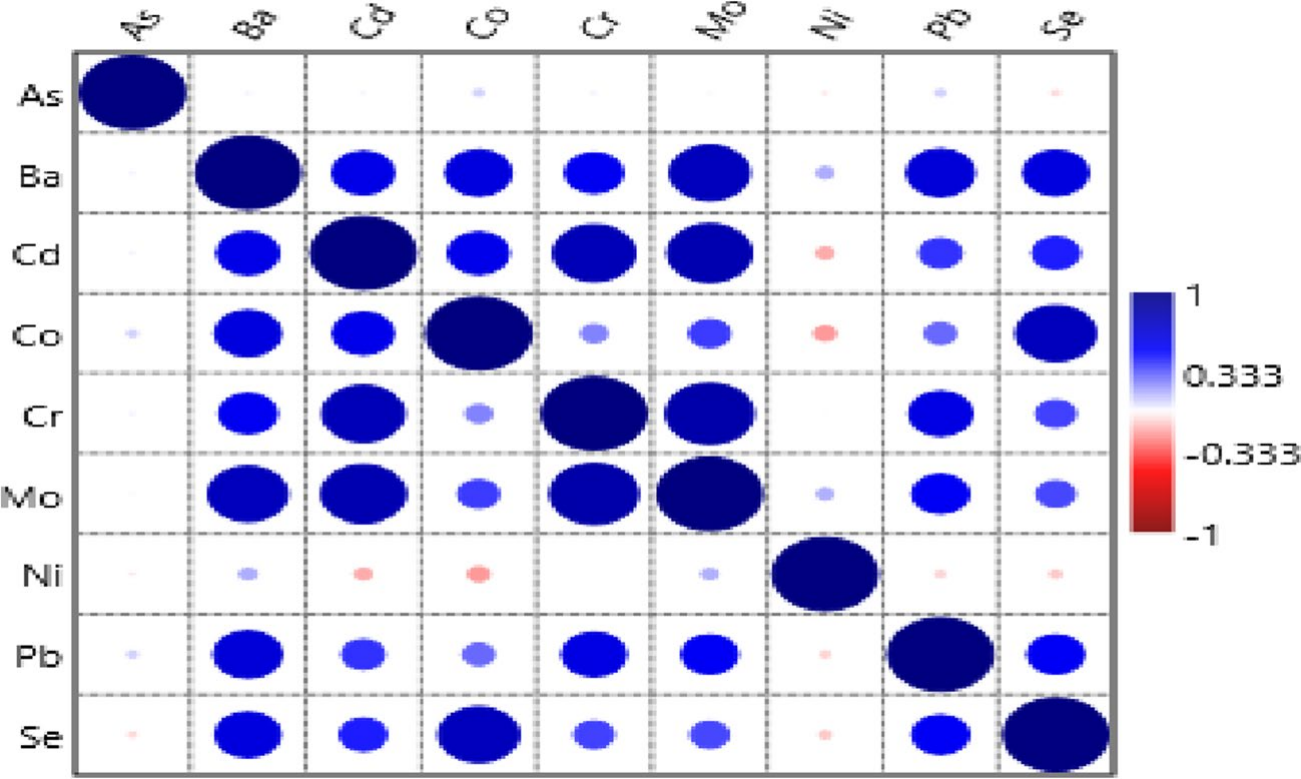
Pearson correlation (r) between heavy metal contents (As, Ba, Cd, Co, Cr, Mo, Ni, Pb and Se) of tropical fruit varieties

Pearson correlation (r) between macro (P, K, Ca and Mg) and micro (Fe, Zn, Cu, Mn and B) nutrient quantities and moisture contents of tropical fruit varieties is given in Fig. 2. As can be seen by examining Fig. 1; Although there are negative relationships between moisture content and nutritional elements of tropical fruits, the relationships between Cu content (r=-0.696**) are significant and moderately strong negative relationships (p<0.05, r=0.30-0.70), P (r=-0.750**). ), K (r=-0.948**), Mg (r=-0.906**) and B (r=-0.965**) contents were found to be significant and highly negative relationships (p<0.05, r>0.70). ) was determined to be. Although there are positive relationships between P contents of fruits and other nutritional elements, there are significant and moderately strong positive relationships with Ca (r=0.574**) and Fe (r=0.606**) contents, K (r=0863**), Mg (r). =0.796**), Cu (r=0.807**) and B (r=0.728**) contents showed significant and high-strength positive relationships. It was determined that there were significant and highly strong positive relationships between the K contents of tropical fruit varieties and their Mg (r=0.957**), Cu (r=0.815**) and B (r=0.945**) contents. Significant and moderately strong positive relationships were determined between the Ca quantities of the varieties and their Fe (r=0.543**) and Cu (r=0.647**) contents, while the Mg quantities of the fruits and their Cu (r=0.774**) and B (r=0.888**) quantities were determined. It has been revealed that there are significant and highly strong positive relationships between contents. In addition, when the Fe and Cu quantities of tropical fruits are examined, it is determined that there is a significant and moderately strong positive relationship between Fe contents and Mn contents (r = 0.666**), and a significant and high strong relationship between Cu contents (r = 0.822**). It was determined by Pearson correlation that there was a significant and moderately strong positive relationship between Cu contents and Mn quantities (r = 0.548**), and a significant and highly strong positive relationship between B quantities and (r = 0701**).

Pearson correlation (r) between heavy metal contents (As, Ba, Cd, Co, Cr, Mo, Ni, Pb and Se) of tropical fruit varieties is given in Fig. 2. As can be seen from examining the correlation between the heavy metal quantities of fruits, there is a positive relationship between the heavy metal quantities of the varieties. While a significant relationship was observed between Ba contents of tropical plants and Cd (r=0.592**), significant relationships were also detected between the contents of Co (r=0.628**), Cr (r=0.553**), Pb (r=0.650**) and Se (r=0.622**) and Mo contents (r= 0.759**). It was stated that the Cd quantities of the fruit varieties showed significant and moderately strong positive relationships with their Co contents (r=0.580**), and significant and high strong positive relationships with their Cr (r=0.779**) and Mo (r=0.803**) contents. In addition, it was found that there were significant and moderately strong relationships between the Cr quantities of tropical fruits and their Pb contents (r=0.607**), while there were also significant and highly strong positive relationships between their Mo contents (r=0.835**). When looking at the Co, Mo and Pb quantities of tropical fruit varieties; It was determined that there were significant and moderately strong positive relationships between the Mo contents of the fruit varieties and their Pb contents (r=0.535**), and the Pb contents with their Se contents (r=0.536**), while the Co quantities were found to have a correlation with their Se contents (r=0.760**). The Pearson correlation analysis conducted in this study aimed to reveal the strength and direction of the relationship between the nutrient element or heavy metal contents of variable tropical fruit varieties. Thus, Pearson correlation tried to draw the best fit line on the data of tropical fruit varieties and nutrient element contents or tropical fruit varieties and heavy metal contents, and the Pearson correlation coefficient (r) revealed how far all these data points were from the best fit line. [24].

## Conclusion

Fruit types have caused changes in the macroelement and moisture contents of tropical and subtropical fruits. Calcium contents of Pineapple and Pepino fruits were found to be at the lowest levels compared to other fruits, and the highest P and K amounts were determined in Gooseberry and Tamarind fruits, respectively. It was revealed that the fruits used in this study are rich in potassium. The highest P amounts were detected in Gooseberry and Tamarind fruits. As a micro element, the most abundant element in fruits was Fe. In general, toxic element quantities of fruits were established at very low levels (except As and Ba). As with macro- and microelements, results regarding toxic concentrations varied depending on fruit type.

## Data Availability

Not applicable.
